# A Pilot Study on Anti-Obesity Mechanisms of *Kappaphycus* *Alvarezii*: The Role of Native κ-Carrageenan and the Leftover Sans-Carrageenan Fraction

**DOI:** 10.3390/nu11051133

**Published:** 2019-05-21

**Authors:** Yao Xian Chin, Ye Mi, Wan Xiu Cao, Phaik Eem Lim, Chang Hu Xue, Qing Juan Tang

**Affiliations:** 1 Human Health Research Laboratory, College of Food Science and Engineering, University of China, Qingdao 266003, Shandong, China; chinyx1@gmail.com (Y.X.C.); MYmy824@163.com (Y.M.); caowanxiu2014@sina.com (W.X.C.); 2 Institute of Ocean and Earth Sciences, University of Malaya, Kuala Lumpur 50603, Malaysia; phaikeem@um.edu.my

**Keywords:** *Kappaphycus*, obesity, metabolic syndrome, carrageenan, food additive, zero-waste, functional food, precision nutrition, gut microbiota

## Abstract

*Kappaphycus* is a commercially important edible red alga widely cultivated for carrageenan production. Here, we aimed to investigate the anti-obesity mechanism of *Kappaphycus* *alvarezii* by comparing the effects of whole seaweed (T), extracted native κ-carrageenan (CGN), and the leftover fraction sans-carrageenan (SCGN) supplementations (5%, *w*/*w*) on diet-induced obese C57BL/6J mice. A high-fat diet induced both a raised body fat percentage and serum cholesterol level, increased adipocytes size, abnormal levels of adipocytokines, and promoted gut dysbiosis. Our results showed that, overall, both CGN and SCGN were more effective in reversing obesity and related metabolic syndromes to normal levels than T. Furthermore, these findings suggested that CGN- and SCGN-modulated gut dysbiosis induced by a high-fat diet, which may play an influencing role in adiponectin dysregulation. Our data also showed some evidence that CGN and SCGN have distinct effects on selected genes involved in lipid metabolism. In conclusion, both κ-carrageenan and SCGN have novel anti-obesity potential with possible different mechanisms of action.

## 1. Introduction

The global prevalence of overweight and obesity has become a major concern for societies around the world and places a great burden on governments and individuals alike, both medically and financially. Even greater, concerted efforts from health authorities in promoting health awareness are not enough to prevent the growth of overweight and obese populations, regardless of the economic status of the society [1]. While exercise and healthy living habits are vital for any weight management regime, diet undeniably plays a large part in sustaining its success. Dietary interventions such as a balanced diet coupled with calorie restriction have been proven to be effective in reversing metabolic complications linked to obesity such as diabetes [2]. However, the reality is that implementing and maintaining calorie restriction in daily life is both challenging and ineffective for many people, as it is susceptible to many disruptive factors including both behavioral and physiological vulnerabilities [3,4,5,6]. Hence, there is a need for daily diets to incorporate food that promotes weight loss. While terrestrial plants have been extensively investigated for anti-obesity compounds, aquatic plants are still underutilized as a potential source of such bioactive compounds, even though marine plants such as seaweeds constitute a significant portion of diets in some cultures.

The red seaweed *Kappaphycus* sp. is cultivated commercially in tropical countries such as the Philippines, Indonesia, and Malaysia, as well as many in East Africa. Though mainly cultivated for its production of the hydrocolloid κ-carrageenan, *Kappaphycus* is also a common food ingredient among local populations and is believed to have various beneficial effects. The seaweed has a high nutrient content [7] and contains significant amounts of phytochemicals [8], many of which were discovered to have anti-obesity potential [9]. A recent study has showed that supplementing *Kappaphycus alvarezii* into feed reduces the effects of a high-fat diet in rats [10]. Nevertheless, the anti-obesity mechanism of *Kappaphycus alvarezii* is still not well defined. The sulfated polysaccharide κ-carrageenan is a major component of *Kappaphycus alvarezii*, accounting for over one-third of its weight [7,11,12], and is considered a dietary fiber. There are numerous studies that describe the nutraceutical and pharmacological potentials of various marine polysaccharides [13,14,15,16,17,18], including carrageenan [19,20,21,22]. Hence, it is plausible that carrageenan is one of the active compounds that are responsible for the obesity resistance property of the seaweed. It must be highlighted that carrageenan is thoroughly reviewed and regarded as safe by major health regulatory bodies despite some researchers linking it to inflammation [23,24,25]. 

In addition, carrageenan processing from *Kappaphycus* would generate about 1 kg of solid waste for every 2 kg of carrageenan produced. Unlike terrestrial plants, the waste constitutes low amount of lignin, which is resistant to enzymatic hydrolysis and chemical degradation and, therefore, more amenable to further downstream processing [26]. Currently, the waste from carrageenan processing industry has been mainly utilized as a fertilizer or soil conditioner. Since the waste would likely contain many of the *Kappaphycus* nutrients, there are a number of studies conducted to research biorefinery methods that would allow the potential utilization of the waste for other purposes including the recovery of valuable bioactives, protein, and carbohydrates [27]. 

Therefore, in this paper we aimed to further elucidate the mechanism behind the weight loss effect of *Kappaphycus alvarezii*. C57BL/6J mice were chosen as an animal model, as they are susceptible to diet-induced obesity and can develop metabolic syndromes similar to ones seen in humans, such as hyperglycemia and hyperinsulinemia [28,29]. We compared the weight loss effect between whole *Kappaphycus* alvarezii (T), native κ-carrageenan (CGN) extracted from the seaweed, and the leftover sans-carrageenan fraction (SCGN, which represents carrageenan processing waste) in overweight C57BL/6J mice by characterizing their impact on the phenotypical and biochemical changes in mice. Furthermore, we examined the changes in gut microbiota in these mice after a substantiate period of treatment.

## 2. Materials and Methods

### 2.1. Sample Acquisition and Processing

Fresh *Kappaphycus alvarezii* were bought from local merchants in Semporna, Sabah, Malaysia. The seaweed were morphologically identified as *Kappaphycus alvarezii* according to features outlined previously [30]. The seaweed were washed thoroughly with tap water to remove mud and debris. Small mollusks and epiphytes were removed using forceps and gentle brushing. Cleaned seaweed were then air-dried in air-drying cabinet at 30 °C for 48 h. Dried seaweed were kept at room temperature in resealable zip-lock bags until use. 

### 2.2. Preparation of Study Diets

Dried *Kappaphycus alvarezii* were milled into powder using a conventional food grinder. Milled seaweed powder was then shifted through a sieve to remove large chunks of seaweed. Carrageenan were extracted from the seaweed powder using parameters as described by Webber et al. [11]. In brief, 10 g of seaweed powder were placed in a filter cloth bag and soaked in 500 mL of reversed osmosis water at 74 °C for 4 h with constant stirring. At the end of the extraction process, the polymerization of carrageenan was induced by the addition of 95% ethanol into the hot mixture at a 3:1 ratio. The polymerized carrageenan was filtered out and repeatedly washed using 95% ethanol until it appeared as a light-yellowish fiber. The carrageenan extraction process was repeated twice to ensure carrageenan were fully extracted out from the seaweed powder. The remaining mixture was condensed using a rotatory evaporator at 45 °C, and any carrageenan residue were washed out using 95% ethanol. The sans-carrageenan mixture was then combined with the water-insoluble fibers in the filter cloth bag and air-dried to obtain the sans-carrageenan fraction. The study diets were then prepared as follow: A normal, low-fat rodent diet (LFD) with 10% kcal energy from fat (D12450J, Research Diets Inc.; Table S1), a high-fat rodent diet (HFD) with 45% kcal energy from fat (D12451J, Research Diets Inc.; Table S2), HFD supplemented with 5% whole *Kappaphycus alvarezii* (T), 5% κ-carrageenan (CGN), and 5% sans-carrageenan fraction (SCGN), respectively. A control HFD supplemented with 5% lipase inhibitor drug Orlistat (O) was used as a comparison.

### 2.3. Animal Study

All the experimental procedures were done in accordance to guidelines published by the National Institutes of Health (Guide for the Care and Use of Laboratory Animals, 8th edition). Approval for animal study was granted by the Committee on the Ethics of Animal Experiments of Ocean University of China (Approved protocol ID SCKK2012-0001). The number of animals required to established a meaningful obesity model, defined as overweight by 20%, was determined by power analysis. Using G*Power, it was determined that five animals per group was enough to achieved statistical power of 0.98, with the Type I error rate set at 0.05.

A total of fifty-four male, specific-pathogen free (SPF) C57BL/6J mice (14–18 g, 4-weeks old) were purchased from Vital River Laboratory Animal Technology Co., Ltd. (Beijing, China). The mice were each individually housed at 23 ± 3 °C on a 12 h light/dark cycle and were provided drinking water (mineral water) and food ad libitum. After a week of acclimatization on standard chow, the animals were separated randomly into six groups (*n* = 9). One group (N) was fed with a LFD while the remaining five groups were fed with HFD for another ten weeks. Feed and drinking water were changed every other day, and beddings (wood shavings) were changed three times a week. The animal house was cleaned daily, and the floor was disinfected with mild detergent every week. The mice were monitored regularly for any physiological and behavioral abnormalities such as dulling of fur, restlessness, and extreme aggression that may be indicators of diseases. At the end of ten weeks, the HFD-fed mice were regrouped randomly into 5 groups (*n* = 9) of different treatments: One group (M) would continue with the HFD, while the others would change into T, CGN, SCGN, and O diets (Table 1). The dietary intervention lasted for six weeks, with fresh feed and water given every two days throughout the experimental period. Daily energy intake and weight changes per week were measured and recorded for each group. On week 15, the mice were subjected to an oral glucose tolerance test (OGTT). Feces and blood samples were collected before the mice were sacrificed on week 16 using cervical dislocation.

### 2.4. Oral Glucose Tolerance Test (OGTT)

Mice were deprived of food with access to water for 12 h before being subjected to an OGTT. In brief, mice were restrained using a cylindrical restrainer. Each mouse was gavaged with a dose of glucose at a concentration of 2 g/kg body weight. Blood samples were collected from the mice via a tiny puncture at the tail vein at time points 0, 30, 60, 90, and 120 min. Once collected, the blood samples were placed at room temperature for 15 min before storing on ice. Sera were then separated out by centrifuge (7500 rpm, 15 mins, 4 °C), and the glucose contents were measured using a commercial colorimetric kit (Biosino Bio-Tech, Beijing, China) according to manufacturer’s instructions.

### 2.5. Phenotype Measurements

Mice were induced into general anesthesia with diethyl ether. Blood samples were collected immediately via orbital sinus and processed to obtain serum, as mentioned previously. The liver, visceral fat (epididymal, retroperitoneal, omental), thymus, and spleen were isolated and weighted. The color and size of liver and colon were observed and recorded. Thymus, spleen (organ weight/body weight), and Lee’s obesity indices (body weight (g)^3 / body length (cm)) were calculated for each mouse. 

### 2.6. Histology

Liver and epididymal adipose tissues were fixed in 4% paraformaldehyde for 3 days, processed, and embedded in paraffin wax. Thin sections (5 μm) were cut and placed on slides. Epididymal adipose tissues were stained with hematoxylin and eosin (H and E), while liver tissues were stained with H and E and oil red. Adipocytes surface areas and diameters were calculate using Adiposoft plugin in ImageJ software.

### 2.7. Sera Biochemical Analyses 

Total cholesterol, high-density-level (HDL) cholesterol, low-density-level cholesterol, triglyceride, and lipase levels in serum were determined using commercial kits (Biosino Bio-Tech, Beijing, China). Circulating adipocytokines, namely adiponectin and leptin, and insulin levels were also measured using ELISA kits (Nanjing Jiancheng Bioengineering Institute). 

### 2.8. RNA Extraction

Total RNA was extracted from tissues using the standard TRIzol protocol [24] with slight modifications. In brief, 50–100mg of frozen hepatic and colon tissues were homogenized in 1 mL of TRIzol reagent at room temperature (RT) using a tissue homogenizer (Bioprep-24 homogenizer, Allsheng). After incubation for 5 min at RT, a volume of 200 μL chloroform was added to the homogenates, and they continued incubate at RT for another 10 min. The samples were then centrifuged (12,000 g, 10 mins, 4 °C). The clear aqueous layer containing the RNA was transferred to new tubes. An equal volume of isopropanol was added to the samples to precipitate the nucleic acids. After being incubated for 10 min, the mixtures were centrifuged (12,000 g, 10 mins, 4 °C). The supernatants were discarded, and the pellet was re-suspended in 1 mL of 75% ethanol. The samples were then centrifuged (7500 g, 5 mins, 4 °C) and air-dried for 5–10 min to obtain pellets of RNA which were re-solubilized in 100 μL of deionized-distilled water. The purity, concentration, and integrity of the extracted RNA were checked using Nanodrop (Thermo Fisher, Waltham, Massachusetts, USA) and agarose gel electrophoresis. 

### 2.9. Real-Time qPCR (RT-qPCR)

A total of 2 ng of RNA was used for synthesis of complementary DNA (cDNA) using 5X All-In-One RT MasterMix (with AccuRT Genomic DNA Removal Kit) (ABM, Vancouver, Canada). The synthesized cDNAs were stored at −20 °C until analyzed using RT-qPCR.

The RT-qPCR was run on a X960 real-time PCR system (Heal Force, Shanghai, China). The qPCR reaction consists of 10 μL of BrightGreen 2X qPCR MasterMix-S (ABM, Vancouver, Canada), 0.6 μL of 10 μM of forward and reverse primers (Table 2), 2 μL of cDNA template, and 6.8 μL of nuclease-free water. The thermal profile included an initial denaturation step at 95 °C for 10 min, followed by forty-five cycles of denaturation at 95 °C for 15 s, annealing at 60 °C for 20 s, and extension at 72 °C for 30 s. A beta-actin gene was used as a housekeeping gene for normalization. The relative expression of sterol regulatory element-binding protein—1a (*srebp-1a*), acyl-CoA oxidase (*aco*), peroxisome proliferator-activated receptor alpha (*ppar*-*α*), fatty acid synthase (*fasn*) and adiponectin receptor 2 (*adipoR2*) in the liver, adiponectin receptor 1 (*adipoR1*) and adipoR2 in the colon, and Niemann-Pick C1-like 1 (*NPC1L1*) in the small intestine were measured using standard curve method. 

### 2.10. ELISA

Protein levels of selected genes in 2.9 that were found to have significant changes in mRNA expression were measured using a commercial ELISA kit (ELSBIO, Suzhou, China) according to the manufacturer’s instructions.

### 2.11. Fecal Short-Chain Fatty Acids (SCFAs)

Fecal samples were retrieved from −80 °C freezer on the day of analysis. About 200 mg of feces were dissolved in 1200 μL of ultrapure (Type III reagent) water (Unique-R20, RSJ Scientific Instruments). After being fully homogenized, 50 μL of sulfuric acid (50%, *v*/*v*) was added to the homogenate and kept at RT for 5 min, with an intermittent vortex every minute. The acidified samples were then centrifuged (5000 g, 10 mins, RT) to obtain a clear supernatant. The supernatant was transferred to new tubes, and 50 μL of 2-ethyl butyrate acid solution (1%, *v*/*v*) were added as internal standard. The mixture was then topped up with 500 μL of diethyl ether and vortexed briefly for 30 s before being centrifuged (5000 g, 10 mins, RT). The clear supernatant was transferred to a new tube. A volume of 1 μL of the supernatant was injected into a gas chromatography (GC) system (Agilent 6890 series, Agilent Technologies) with a GC column (DB-FFAP, Agilent) using methods established previously [25]. 

### 2.12. Gut Microbiota Profiling

Fecal DNA from group N, M, SCGN, and CGN were extracted using a QiaAmp DNA Stool Mini Kit (Qiagen, cat. no. 51604) according to manufacturer’s protocol, and DNA integrity was verified using gel electrophoresis. Gut microbiota profiling was performed by using 16S rRNA analysis. The V3–V5 region of the 16S rRNA gene was selected for amplification. Sequencing was done by a genomic service provider, Novogene, on 5 samples from each group. Briefly, the total genome DNA was extracted from samples using the CTAB/SDS method, and the purity and integrity were evaluated on 1% agarose gel. 16S rRNA/18S rRNA/ITS genes of distinct regions (16S V4/16S V3/16S V3–V4/16S, V4–V5, 18S V4/18S V9, ITS1/ITS2, Arc V4) were amplified used specific primers (e.g., 16S V4: 515F–806R, 18S V4: 528F–706R, 18S V9: 1380F–1510R, etc.) tagged with barcodes. All PCR reactions were carried out with the Phusion^®^ High-Fidelity PCR Master Mix (New England Biolabs, Ipswich, MA, USA). Sequencing libraries were generated using the Ion Plus Fragment Library Kit (Thermo Scientific, Waltham, Massachusetts, USA), following the manufacturer’s recommendations. The library quality was assessed on the Qubit@ 2.0 Fluorometer (Thermo Scientific, Waltham, Massachusetts, USA). At last, the library was sequenced on an Ion S5TM XL platform, and 400 bp/600 bp single-end reads were generated. The reads were then split and filtered, and chimeras were detected using the UCHIME algorithm before being removed, obtaining effective tags of sequences. The sequences were analyzed and annotated using Uparse software (Uparse v7.0.1001) against the SILVA database. The created operational taxonomic unit (OTU) table was then subjected to various analyses, including alpha- and beta-diversity indices, LEfSe (Linear discriminant analysis Effect Size) with the threshold log LDA score set at 4.0, and Spearman correlation with selected phenotype and biochemical characteristics of mice.

### 2.13. Statistical Analysis

All values in-text are presented as mean ± standard deviation (SD). Statistical significances were determined by analysis of variance (ANOVA) with Tukey’s or Dunnett’s post hoc test where appropriate using, GraphPad Prism 7 and PASW Statistics 18 software. All figures were constructed using GraphPad Prism 7, Explicet, and the i-Sanger platform.

## 3. Results

### 3.1. Carrageenan (CGN) and Sans-Carrageenan (SCGN) Normalized Animal Obese Phenotype More Than Whole Kappaphycus (T)

**Figure 1:**
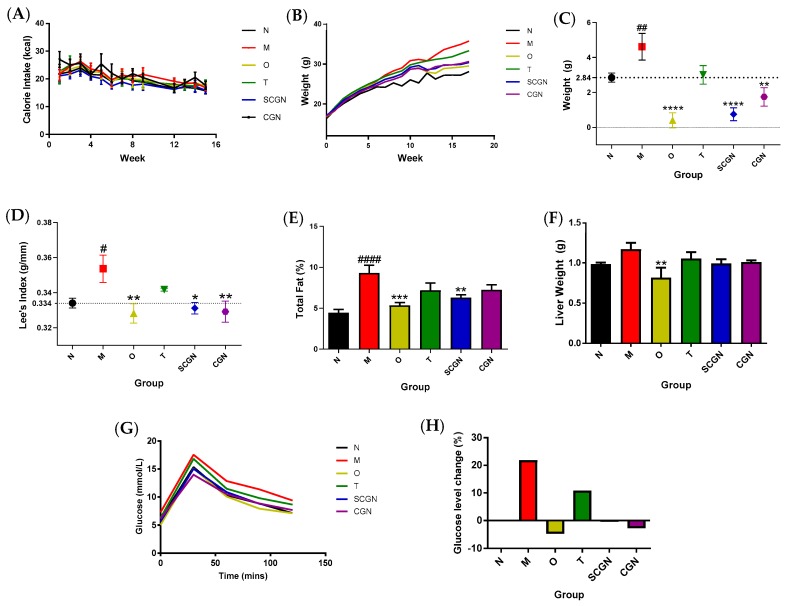
The effects of diet on phenotype of overweight C57BL/6J mice: (**A**) Energy intake throughout the experiment, (**B**) body weight fluctuations, (**C**) accumulated weight changes after dietary intervention, (**D**) Lee’s obesity index, (**E**) total fat percentage, (**F**) percentages of liver weight changes relative to N, (**G**) oral glucose tolerance test (OGTT), (**H**) AUC of OGTT relative to N. N = Normal, M = Model, O = Orlistat, T = Whole *Kappaphycus*, CGN = Carrageenan, SCGN = Sans-carrageenan fraction. Values are given as mean ± SEM, n = 7–8. Statistical significances were calculated using one-way or two-way ANOVA with Tukey post-test where appropriate (* and # *p* < 0.05, ## and ** *p* < 0.01, *** *p* < 0.0005, #### and **** *p* < 0.0001); # denotes versus N, * denotes versus M.

In order to evaluate the anti-obesity potential of *Kappaphycus* and its components, we first compared the effects of T, CGN, and SCGN on the phenotypical changes in overweight C57BL/6J mice after six weeks of treatment. No abnormal physiological and behavioral changes were observed. There was no significant difference in energy intake between the groups throughout the experiment (Figure 1A), suggesting that the appetite of mice was relatively unaffected by the additions of supplements in their feed. All treatment diets successfully reduced the effect of the HFD in terms of weight gained, Lee’s obesity index, total fat percentage, and liver weight (Figure 1). In terms of weight loss (Figure 1B,C), group SCGN showed the most drop in accumulated weight gain during the six weeks of treatment, with a mean of 0.75 ± 1.05 g weight gained, which is comparable to effect of orlistat intake (group O, mean = 0.40 ± 1.20 g). Carrageenan supplementation (group CGN) also caused a significant decrease in weight gain (1.74 ± 1.50 g). However, whole seaweed supplementation (group T) did not elicit significant weight loss (3.00 ± 1.50 g) compared to model (4.61 ± 2.16 g). The leanness of the mice was indicated by Lee’s index (Figure 1D), which showed that both SCGN (0.331 ± 0.008) and CGN (0.329 ± 0.015) achieved scores comparable to mice in group N (0.334 ± 0.007). The total fat percentages of mice were significantly reduced in SCGN (6.30 ± 0.91%) compared to M (9.31 ± 2.48%), although CGN (7.26 ± 1.60%) were not. Nevertheless, the visceral fat of the mice was visibly reduced in both group CGN and SCGN compared to the model, group M, while the decrease in group T was less noticeable (Figure S1). The liver weights in treatment groups, although not statistically significant, were also more aligned to normal compared to model overweight mice (Figure 1F), while the oil red staining of hepatic tissues revealed visually fewer fat droplets in CGN and SCGN but not in the T group (Figure S2). The thymus and spleen indices were not significantly different between groups (Figure S3), suggesting no inflammation occurred. Moreover, mice on the HFD (group M) displayed significant elevated blood glucose levels (Figure 1G) compared to mice on the LFD (21.8% increase) during the OGTT (Figure 1H), whereas supplementation with CGN and SCGN in the HFD were shown to return to levels comparable to group N. However, supplementation with whole *Kappaphycus* only partially improved the blood glucose level (10.9% increase from group N; Figure 1H). 

**Figure 2:**
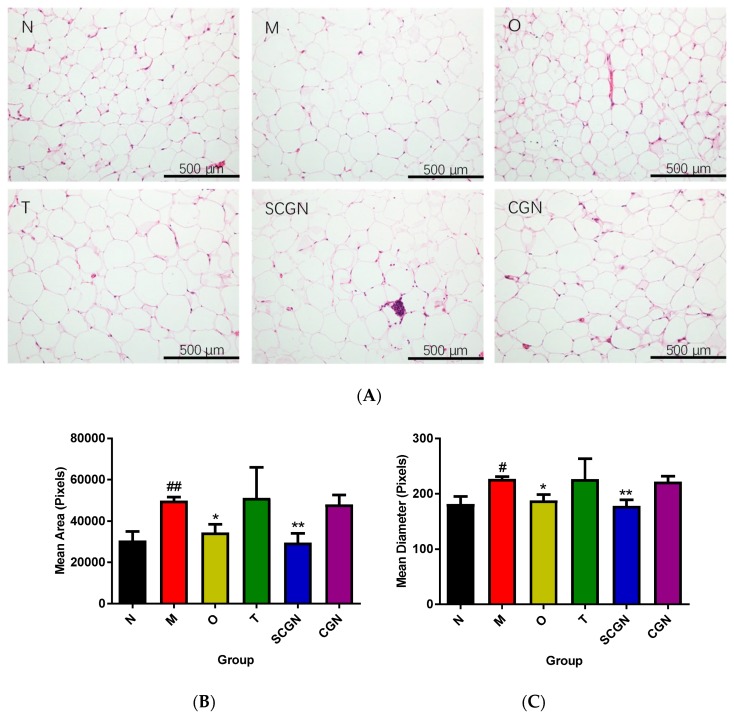
(**A**) Histology (200× magnification), (**B**) size, and (**C**) diameter of adipocytes in mice. N = Normal, M = Model, O = Orlistat, T = Whole *Kappaphycus*, CGN = Carrageenan, SCGN = Sans-carrageenan fraction. Values are given as mean ± SEM, *n* = 7. Statistical significances were calculated using one-way ANOVA with Tukey post-test (# and * *p* < 0.05, ## and ** *p* < 0.01); # denotes versus N, * denotes versus M.

As the metabolic functions and activities of adipocyte changes with size [31], we proceeded to measure the adipocyte size and diameter. Visually, groups N, O, CGN, and SCGN displayed a considerable reduction in adipocyte size (Figure 2A). However, after calculation by Adiposoft, only group SCGN was deem to achieve a significant reduction in adipocytes size, returning to approximate size in group N (Figure 2).

### 3.2. Dietary Treatments Improved Sera Biochemical Profile of Mice

**Figure 3:**
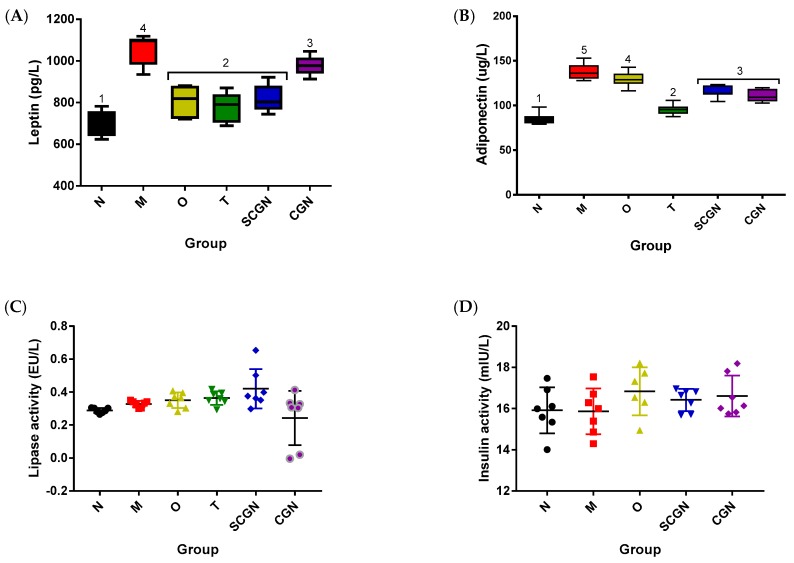
Biochemical analysis of mice sera: (**A**) Leptin level, (**B**) Adiponectin level, (**C**) Lipase level, (**D**) Insulin level. N = Normal, M = Model, O = Orlistat, T = Whole *Kappaphycus*, CGN = Carrageenan, SCGN = Sans-carrageenan fraction. Values are given as mean ± SEM, *n* = 7–8. Statistical significances were calculated using one-way ANOVA with Tukey HSD test (numbers denote corresponding homogeneous subsets).

Following conformation that dietary interventions were successful in restoring the normal phenotype in obese mice, we further analyzed the sera levels of lipid and adipocytokines. The serum levels of total cholesterol, triglycerides, and low-density lipoprotein (LDL)-cholesterol were all partially restored to normal levels in all treated mice, even though the reductions were not statistically significant (Figure S5). On the other hand, the high-density lipoprotein (HDL)-cholesterol of all treatment groups were increased, although only group SCGN (3.07 ± 0.83 mmol/L) reached statistically significant difference from group M (2.02 ± 0.82 mmol/L; Figure S4C). Even though the triglyceride (TG) levels were not significantly different between all groups, both CGN (1.63 ± 0.34 mmol/L) and SCGN (1.62 ± 0.07 mmol/L) nonetheless displayed a moderate decrease from M (1.82 ± 0.38 mmol/L) (Figure S5B). Interestingly, both adipocytokines leptin and adiponectin showed significant increases in HFD-fed mice (1052.0 ± 71.80 pg/L and 137.70 ± 9.10 μg/L, respectively; Figure 3A,B). However, all dietary treatments partially returned the levels towards normal (701.10 ± 64.69 pg/L and 85.35 ± 6.51 μg/L, respectively), with group T (775.6 ± 72.12 pg/L and 95.79 ± 5.85 μg/L) showing the most effect. Both serum insulin and lipase levels were not significantly altered by the dietary changes (Figure 3C,D). Serum insulin was not measured for group T, as its OGTT result did not warrant a further investigation on glucose metabolism.

### 3.3. Different Influences of CGN and SCGN on Gene Involved in Lipid Metabolism

**Figure 4:**
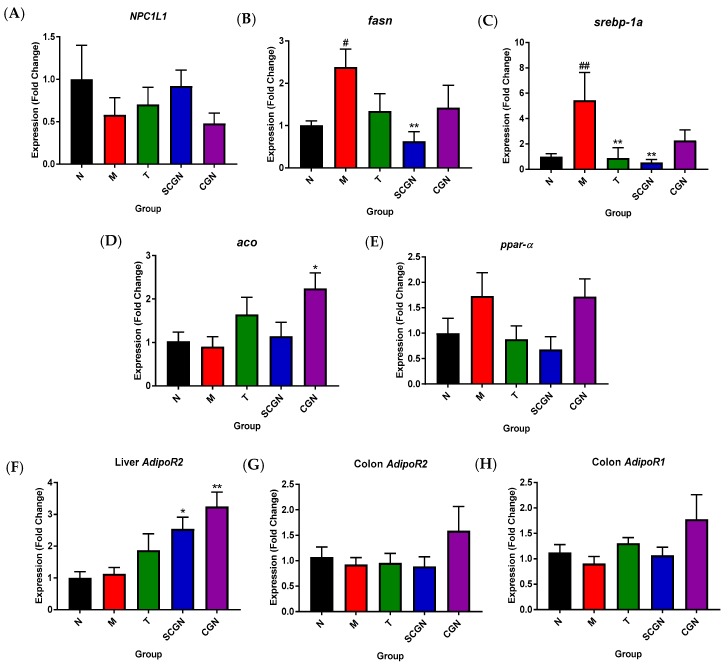
Gene expressions as measured using RT-qPCR: (**A**) *NPC1L1* expression in small intestine; (**B**) *fasn,* (**C**) *srebp-1a,* (**D**) *aco,* (**E**) *ppar-α*, and (**F**) *adipoR2* expressions in hepatic tissues; (**G**) *adipoR1* and (**H**) *adipoR2* expressions in colon. N = Normal, M = Model, T = Whole *Kappaphycus*, SCGN = Sans-carrageenan fraction, CGN = Carrageenan. Values are given as mean ± SEM, *n* = 7–8. Statistical significances were calculated using one-way ANOVA with Tukey post-test (* and # *p* < 0.05, ## and ** *p* < 0.01); # denotes versus N, * denotes versus M.

Since the lipid profile in T, CGN, and SCGN groups generally showed a downward trend, we further investigated the dietary effects on the expression of selected genes in lipid metabolism pathway (Figure 4). The measurement of the lipid transporter (*NPC1L1*) mRNA expression in the small intestine did not differ significantly between model and treatment groups, suggesting lipid intake were comparable between groups. We then measured the gene expression for lipid metabolism. Compared to the N group, the M group displayed a significantly higher expression for genes involved in lipid synthesis (mean 5.45-fold and 2.39-fold of N for *srebp-1a and fasn*, respectively) but not for other parameters except for *ppar-α* (mean 1.73-fold of N), which was slightly higher but not significant. Genes for lipid synthesis, *srebp-1a* and *fasn* were reduced in the liver of all treatment groups, with SCGN being the most significant (mean 0.54-fold and 0.63-fold of N, respectively). On the other hand, the lipid metabolism gene *aco* mRNA expression was also higher in treatment groups, although the increment was only significant for group CGN (mean 2.47-fold of N). Group CGN also demonstrated a higher, albeit not significant, mRNA expression of adiponectin receptors (*adipoR1* and *adipoR2*) in colonic tissue, although all three treatments were shown to have mild-to-moderate incremental effect on *adipoR2* expression in hepatic tissue, with both SCGN and CGN showing significance at *p* < 0.05 and *p* < 0.01, respectively. Additionally, only CGN maintained the relatively high PPAR-α expression (mean 1.72-fold of N) level seen in HFD-fed mice (1.73-fold of N). 

**Figure 5:**
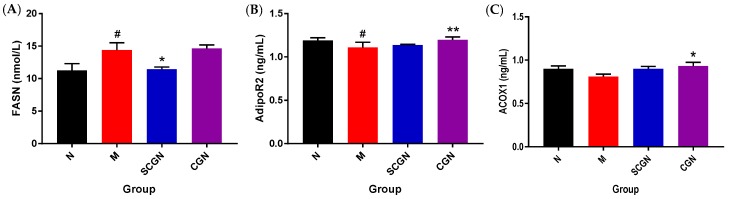
Protein levels of selected genes with significant changes in mRNA expressions: (**A**) fatty acid synthase (FASN), (**B**) AdipoR2, and (**C**) ACO hepatic levels. Values are given as mean ± SEM, *n* = 5. Statistical significances were calculated using two-way ANOVA with Dunnett post-test (* *p* < 0.05, ** *p* < 0.01); # denotes versus N, * denotes versus M.

Selected gene expressions that were significantly different (fasn, aco and hepatic adipoR2) after treatment were then measured for their protein levels (Figure 5) using ELISA kits. For SCGN, the level of hepatic fatty acid synthase (FASN) was significantly reduced, while the levels of hepatic adipoR2 and acyl-CoA oxidase (ACO) were not significantly different from M, although the level of adipoR2 did show minute increment (≈2.6%). On the other hand, CGN increased the level of both hepatic adipoR2 and ACO, while its FASN remained unchanged from M. Taken together, these results showed consistency with the measured mRNA expressions. 

### 3.4. CGN and SCGN Changed Fecal SCFA Compositions in Mice

**Figure 6:**
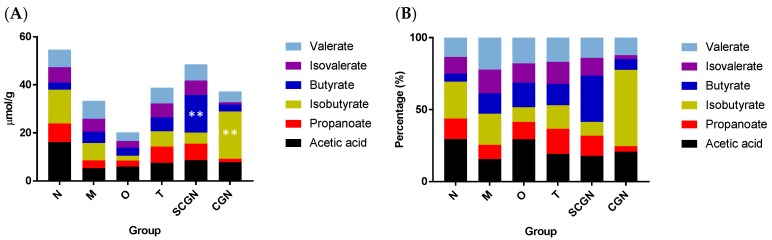
Measurements of total fecal short-chain fatty acids (**A**) and proportion of individual fatty acids (**B**) in mice and the isobutyrate level of CGN compared to M. N = Normal, M = Model, T = Whole *Kappaphycus*, SCGN = Sans-carrageenan fraction, CGN = Carrageenan; Statistical significances were calculated using two-way ANOVA with Dunnett post-test (** *p* < 0.01), *n* = 8.

Besides lipid metabolism, we also hypothesized that the diets would induce changes in the production of short-chain fatty acids (SCFAs) due to the presence of the soluble fiber in CGN and the insoluble fibers in SCGN. Fecal SCFAs analysis (Figure 6) showed that dietary intervention using *Kappaphycus alvarezii* and its component significantly improved the production of SCFAs. The SCGN diet had the most profound effect among all treatments, restoring the total SCFAs level to near normal level (Figure 6A; 48.56 μmol/g compared to 54.69 μmol/g). Though both the CGN and SCGN diets preferably increased the presence of a single fatty acid (Figure 6B; 52.99% or 19.75 μmol/g of isobutyrate and 32.17% or 15.62 μmol/g butyrate, respectively), the increase in group CGN was accompanied by substantial decrease in other types of SCFAs (Figure 6B), namely propanoate (1.39 μmol/g) and isovalerate (1.41 μmol/g), resulting a comparatively low total SCFAs level of 37.27 μmol/g (Figure 6A). In contrast, group T demonstrated a balance distribution of SCFAs production, with a total production slightly higher than group M (Figure 6A; 38.84 μmol/g compared to 33.30 μmol/g). 

### 3.5. CGN and SCGN Modulate Gut Microbiota with Links to Adipocytokines Levels

**Figure 7:**
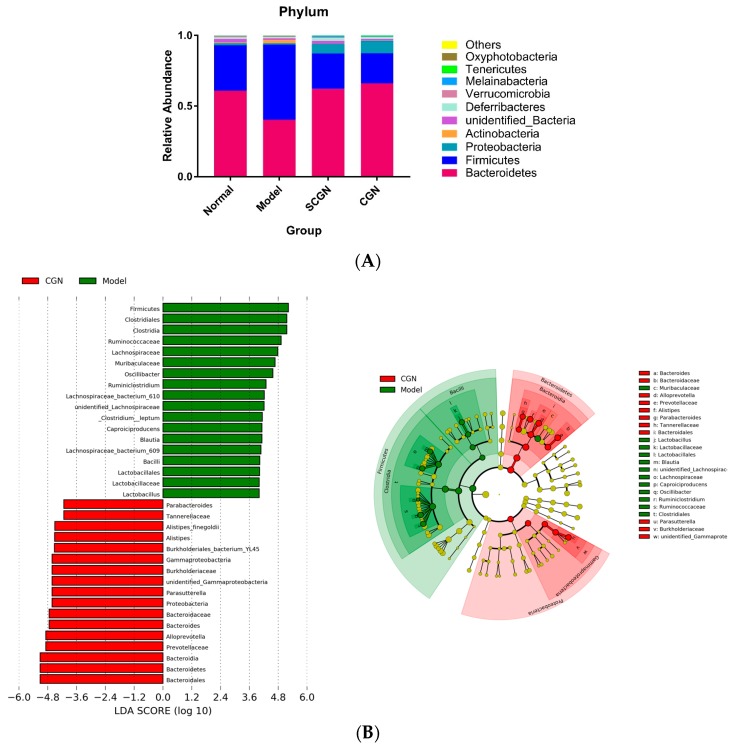

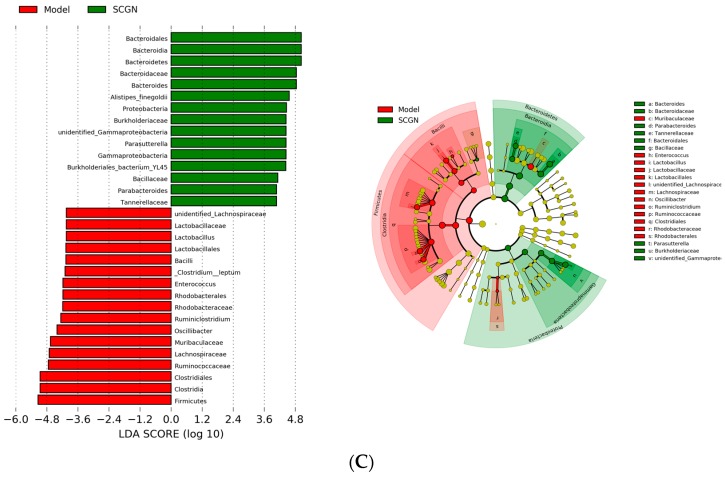
Gut microbiota profile of mice after dietary treatment: (**A**) Major phyla in N, M, SCGN, and CGN, (**B**) LEfSe analysis of CGN versus M, and (**C**) LEfSe analysis of SCGN versus M. N = Normal, M = Model, SCGN = Sans-carrageenan fraction, CGN = Carrageenan; *n*= 5.

Following the detection of SCFAs changes, we further postulated that gut microbiota profile was substantially altered in the CGN and SCGN groups, but not in group T. Gut microbiota profiling using 16S RNA in groups N, M, SCGN, and CGN revealed that treatment with CGN and SCGN diets elicited significant shifts in microbial composition (Figure S6A). The alpha diversity indices, however, pointed to a higher diversity in microbiota in group M (Figure S6B). Both the Bray–Curtis dissimilarity and Euclidean principle component analysis (PCA) showed distinct differences in the gut microbiome of CGN and SCGN from both the normal and model groups (Figure S6C,D). At the phylum level, the most dominant bacteria in all groups were Bacteroides and Firmicutes, with both the CGN and SCGN diets restoring the Bacteroides-to-Firmicutes ratio to levels identical to group N (Figure 7A). Members of the predominant phylum Bacteroides, such as the family Prevotellaceae and the genus *Alistipes*, were significantly increased in both treatment groups. 

**Figure 8:**
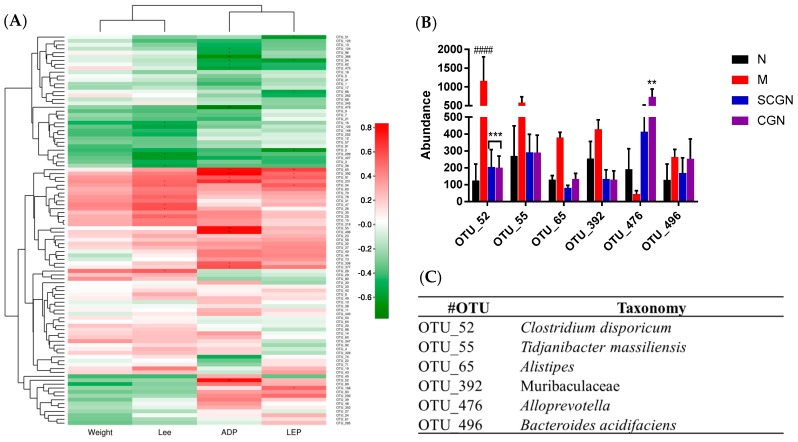
Influence of gut microbes on phenotype and biochemical characteristics: (**A**) Heatmap showing Spearman correlation rank between gut microbes with weight, Lee’s obesity index (Lee), adiponectin (ADP), and leptin (LEP) levels of mice; (**B**) abundance of operational taxonomic units (OTUs) correlated adiponectin levels in mice; (**C**) table showing the taxonomy classification of OTUs in (**B**). Pearson correlation ** *p* > 0.6, *** *p* > 0.75. Statistical significances were calculated using one-way ANOVA with Tukey post-test where appropriate (** *p* < 0.01, *** *p* < 0.0005, #### *p* < 0.0001); # denotes versus N, * denotes versus M.

One of the most striking changes detected by the LEfSe analysis was the relatively low abundance of the known probiotic bacteria *Lactobacillus* sp. in both treatment groups, with a reduction of 20.3-fold and 13.6-fold for group CGN and SCGN, respectively, compared to group M. Other notable changes in the treatment groups include a decrease in abundance of Clostridia, Erysipelotrichaceae, Blautia, and Lachnospiraceae, and an increase in *Parasutterella*, *Alloprevotella*, *Oscillibacter*, Melainabacteria and *Butyricimonas* (Figure S7). Furthermore, four parameters (weight, Lee’s obesity index, serum leptin, and adiponectin levels) were analyzed for correlation with gut microbiota. The results demonstrated that the changes in certain OTUs seemed to correlate with the adiponectin levels in the sera (Figure 8). 

## 4. Discussion

Dietary intervention has been a major strategy in combating global obesity. Nutritional guidelines are regularly issued and revised by numerous authorities and health organizations in line with new discoveries [32]. However, the effects are often not straightforward, with factors such as genetics, physiology, psychology, environments, economics and, more recently, gut microbiomes all influencing the outcome to some degree, resulting in individualized response to otherwise same food ingredients [33,34,35,36]. This is further complicated by the regularity of processed food in an urbanized daily diet, which undergoes various processing and the addition of food additives that would influence both the quality and bioavailability of nutrients [37,38,39,40]. Concerns about food additives have fueled the demand for natural food besides researching on novel properties of existing food. Yet, food additives remain an important, as they provide many functions and enhancements in food that are irreplaceable [41].

In this study, we aimed to explore and define the beneficial effects of *Kappaphycus* sp. and its components, including the common food additive κ-carrageenan, towards the development of obesity. It has been reported that addition of whole *Kappaphycus* in an HFD prevents subsequent metabolic syndrome in Wistar rats [10]. Here, we described the reverse of obesity in overweight C57BL/6J mice after supplementation of *Kappaphycus* (T), carrageenan (CGN), and sans-carrageenan (SCGN) fractions, respectively, in an HFD. Overall, the weight and fat proportion of the animals decreased after treatment, with CGN and SCGN in general having more pronounced weight loss, fat loss, and reduced weight gain compared to whole *Kappaphycus*. All treatments successfully improved the metabolic profile of the HFD-fed mice in varying degrees, although only SCGN successfully returned adipocytes to normal size. Results from the OGTT suggested a possible anti-hyperglycemia property for the CGN and SCGN, which may be due to the increase, albeit insignificant, in the circulating insulin level. Since the energy intake remained relatively constant throughout the feeding period, appetite was thus ruled out as a factor for the improvement of the metabolic syndrome observed. Crucially, the effects on the physical appearances of mice exhibited by CGN or SCGN were better than whole *Kappaphycus*, suggesting that their mechanism were neither synergistic nor cumulative to each other.

This study showed that the serum lipid profile of mice treated with *Kappaphycus*, carrageenan, and sans-carrageenan fractions were all improved. Interestingly, CGN and SCGN were both better individually than whole *Kappaphycus*, and this is evident in the smaller sizes of adipocytes in these two groups compared to group T. As adipocytes are central to obesity pathology, we measured two adipocytokines, leptin and adiponectin, levels in the mice. Adiponectin and leptin play major roles in energy homeostasis, insulin sensitivity, lipogenesis, and lipolysis [42,43]. Leptin is a potent appetite inhibitor, and defects to its protein has been linked to development of obesity [44]. In our study, the leptin level was significantly higher in group M, suggesting leptin resistance, which is associated with diet-induced obesity [45]. Intriguingly, the level of adiponectin was also unexpectedly high in group M. It might be that with the increase in adipocytes, more adiponectin was secreted as well, but its functional effects were offset by the relatively lower mRNA expression of adiponectin receptors adipoR1 and adipoR2 in group M compared to CGN and, to some degree, SCGN. These receptors have been recently proposed of having significant role in obesity and pleiotropic biological traits via its downstream signaling of diverse and complex pathways [46]. Our data demonstrated that CGN partially increased the mRNA expression of *adipoR1* and *adipoR2* in the colon, and while both CGN and SCGN increased adipoR2 in hepatic tissues, only CGN was significant. Thus, higher receptor availability might explain the lower concentration of adiponectin in sera of leaner mice in the CGN groups, although the minute increment in hepatic adipoR2 may also be a part factor in SCGN mice. Based on the results, it would be interesting to study the impact of CGN and SCGN on adiponectin receptors levels in a cell culture model to elucidate their relationships.

Considering that the fat composition, adipose size, total cholesterol, and LDL:HDL ratios were all either reduced or improved in the treatment groups, we further investigated the effects of *Kappaphycus*, carrageenan and sans-carrageenan fractions on lipid metabolism and absorption. The mRNA expression of gene *NPC1L1*, which encodes for a protein critical for intestinal absorption of cholesterol, was identical for every group, suggesting that lipid absorption was not elevated by the diets, which agreed with assessment reached by Wanyonyi et al. [10]. Lower *srebp-1a* and *fasn* expression levels suggest that SCGN primarily achieved its anti-obesity effects via reduced cholesterol production, while CGN appeared to have a different anti-obesity mechanism by showing elevated *aco* expression, which promotes peroxisomal β-oxidation of fatty acids. Moreover, it is possible that CGN also raised metabolism in colonic tissues as evident by the increased expression levels of *adipoR1* and *adipoR2*, which could mediate AMPK and PPAR-α ligand activities, including lipogenesis [47,48]. Crucially, the protein levels of FASN, adipoR2, and ACO in hepatic tissues were also consistent with the mRNA expression results of these genes, giving further credence to our hypothesis. PPAR-α plays a central role in β-oxidation of lipids and is often thought to be upregulated in lean subjects [49]. In contrast, the mice in groups M and CGN our study had relatively high levels of PPAR-α expressed in their livers compared to the mice on the LFD. It could be that both groups of mice continued to utilized fat as their main fuel source, whereas mice on the T and SCGN diets both reduced their reliance on lipid metabolism for energy. Altogether, it could be CGN and SCGN achieved their weight loss effects primarily through separate means, with CGN mainly increased the lipid metabolism, while SCGN reduced fatty acid synthesis, which could be due to smaller adipocytes [31]. It is interesting to note that RT-qPCR results of lipogenic and lipolysis genes in group T displayed a pattern that was not completely aligned to either CGN or SCGN, suggesting that the two fractions of *Kappaphycus* are not synergistic in the modulation of lipid metabolism, as with the scenario in glucose metabolism, but rather are likely to be involved in a series of complex interactions which reduced its overall efficiency. 

Many studies have linked SCFAs to gut health, which in turn is an indicator for gut inflammation that is frequently linked with obesity [50]. Our results showed that the SCFAs of the HFD were partially restored when fed with whole *Kappaphycus*, carrageenan, and SCGN. Butyrate was often associated with better gut health and metabolism [51,52], and it and its isoform, iso-butyrate, were found to be significant higher in proportion SCGN and CGN group, respectively. This is in contrast with the T group, which increased all SCFAs equally, suggesting that each promote growth of different gut bacteria. As SCFAs are produced by gut microbiota and are linked to diets [50], we further investigated the effects of CGN and SCGN on intestinal flora. Studies have shown that gut microbiome plays an important role in metabolic syndrome of obesity [53] by utilizing dietary fibers as energy source and producing beneficial metabolites [54,55]. Both diet and host genotypes are reported as determinants in shaping gut microbiome [56,57]. As *Kappaphycus* is rich in dietary fibers, and both it and carrageenan are reported to influence gut microbiome [10,58], we subsequently investigated the effects of the CGN and SCGN towards gut intestinal flora of the animals to see if the anti-obesity effects were mediated by changes in gut microbiota. An examination of 16S rRNA data revealed that all groups have distinct microbiota from each other, and the feeding of CGN and SCGN did not alter the microbiota composition towards those of group N but rather formed similar compositions away from both M and N diets, as inferred in the PCA plot and Bray-Curtis dissimilarity index plot. Though obesity was often linked to a less diverse gut microbiome, the results of alpha diversity indices in our study implied that the microbial richness of group M was higher than the rest, which could be a direct effect of enrichment of otherwise obscure species by the HFD. Further analysis of 16S sRNA data showed that both CGN and SCGN increased the Bacteroidetes-to-Firmicutes ratio, consistent with lean phenotype reported elsewhere [59]. The abundance of Melainabacteria, a phylum that is associated with a vegetarian diet and predicted to be able to ferment various carbon compounds such as hemicellulosic compounds [60] was also significantly increased in SCGN and, to a lesser extent, in the CGN group, which could explain the higher content of butyrate in SCGN. The family Prevotellaceae, was found to be significantly more abundant in CGN and SCGN. One of its members, *Alloprevotella*, is reported to be negatively correlated to obesity, diabetes, and metabolic syndromes [61,62], and is also a known butyrate producer, which, alongside increased other butyrate producers such as *Butyricimonas*, would contribute to the relatively high levels of isobutyrate and butyrate observed in CGN and SCGN diets. Several studies have indicated that the anti-inflammatory properties of butyrate producers are mediated via butyrate production [63,64]. Another intriguing genus, *Parasutterella*, was also found to be abundant in both CGN and SCGN diets. This bacterium was recently linked to reduced fat intake without decrease in energy intake [65], which agrees with our findings in this study. Besides, *Parasutterella* was reported to increase in diet-induced obesity (DIO) mice when an HFD was replaced with normal chow [66], as was the case in our study. The genus *Alistipes*, another member of Bacteriodetes that was increased in CGN and SCGN diets, was described to have negative correlation with hepatic and serum lipid profile besides being decreased under inflammatory conditions [67,68]. Conversely, CGN and SCGN diets reduced the abundances of bacteria associated with obesity, metabolic syndromes, and inflammation, such as *Blautia*, Lachnospiraceae, and Erysipelotrichaceae [61,69,70] in HFD-fed mice. However, our study also showed that these supplements also greatly reduced the abundance of *Lactobacillus* sp., a known probiotic which is generally regarded as beneficial to intestinal health, which could be due to increased competition from other dominant commensal bacteria such as *Prevotella* [71]. Interestingly, we found that *Clostridium disporicum*, *Alistipes*, *Tidjanibacter massiliensis*, *Bacteroides acidifaciens,* and Muribaculaceae were positively correlated to adiponectin levels in the mice (Figure 8C), while *Alloprevotella* was negatively correlated. Yet, it is still unclear if these relationships were a direct response or cause to physiological changes. Though some bacteria were reportedly linked with adiponectin levels [70,71], the association of the gut microbes found in our study with adiponectin level was not described elsewhere previously, to the best of our knowledge. 

Overall, the changes in gut microbiota were largely in line with the phenotype. While some differences existed between CGN and SCGN microbiota, as evident by the Venn diagram and the Bray-Curtis dissimilatory index, they were not of the major OTUs in their respective population. Therefore, the most changes in microbiota were more likely to be a result from change in host physiology rather than the cause. Nevertheless, considering that both carrageenan and SCGN is unlikely to be absorbed or metabolized by human body [25], it is possible that their anti-obesity effects were mediated by gut microbiota, but these microbes may be relatively obscure in quantity that were not detected by 16S RNA analysis. Hence, adopting a shotgun metagenomics approach coupled with transcriptomics and metabolomics analysis might be able to identify and confirm gut bacteria and their metabolic products that influence the host metabolism seen in our study. In view of this, further studies with a larger sample size and longer feeding period is needed to fully clarify the relationship between CGN and SCGN with gut microbiota. Given that gut microbiota impacts host physiology and vice versa [56,57], it is essential that subsequent research involve pairing of posteriori with a priori microbiome data to help navigate the question whether gut microbiota is a cause or consequences of CGN and SCGN feeding.

Being a key food additive and emulsifier in the food and beverage industry with a reported global production value of USD 762.35 million in 2016 [72], the seaweed hydrocolloid κ-carrageenan has been an important research target for its role in dietary practices. Though concerns about carrageenan consumption have been raised by certain researchers who allege that it causes inflammation and contributes to or promotes colitis [24,73], many experts have questioned the methodology in some of these studies [74,75]. Crucially, the results from these experiments were not replicable in other labs [74,76]. In our study, the feeding of carrageenan did not elicit any signs of colitis and inflammation which was confirm with thymus and spleen indices (Figure S3); and in H and E staining of colonic tissues (Figure S4). The complicated nature of carrageenan and its interactions with other food ingredient presented a challenge for many researchers when interpreting the experimental data, which was outlined and discussed in length by Weiner et al. [77,78]. Henceforth, even though our data showed that the energy intake in mice did not changed with intake of carrageenan, a future experiment to investigate the fate of carrageenan after ingestion is required to determine if supplementation of carrageenan to a diet would change the bioavailability of the nutrients to the host. 

In this climate of zero-waste, scavenging valuable compounds from food processing waste is a constructive method to maximize the use of waste. Here, we also described the potential health benefits of the *Kappaphycus* after carrageenan extraction. The fraction SCGN mainly consisted of insoluble fibers, non-polar molecules, and some water-soluble compounds which were recovered during rotatory evaporation. It would be interesting to find if the anti-obesity effects of SCGN were due to the high fiber content or other compounds which were extracted along with carrageenan.

## 5. Conclusions

In conclusion, *Kappaphycus* has anti-obesity potential which is facilitated by its major soluble fiber, carrageenan, and other compounds through different means. The anti-obesity mechanism is possibly mediated via lipid metabolism, i.e., both lipolysis and lipogenesis. Both CGN and SCGN caused similar changes to murine gut microbiota composition that was different from mice fed with both a LFD and a HFD. Results from this study suggested potential novel applications for carrageenan and carrageenan processing waste, as health food supplement and utilizing a zero-waste strategy in carrageenan production. Our research demonstrated that further investigation into *Kappaphycus* and its components as health food is warranted with focus on their effects on adiponectin production.

## Figures and Tables

**Table 1:** Treatment groups for animal study.

Group	Diet Composition
Normal (N)	10% LFD
Model (M)	45% HFD
Orlistat (O)	45% HFD + 5% Orlistat
Whole *Kappaphycus alvarezii* (T)	45% HFD + 5% whole *Kappaphycus alvarezii*
Carrageenan (CGN)	45% HFD + 5% CGN
Sans-carrageenan (SCGN)	45% HFD + 5% SCGN

LFD = Low-fat diet, HFD = High-fat diet.

**Table 2:** Primer sequences for RT-qPCR.

Gene	Forward Primer	Reverse Primer
*NPC1L1*	GCTTCTTCCGCAAGATATACACTCCC	GAGGATGCAGCAATAGCCACATAAGAC
*AdipoR1*	AGAGCATCTTCCGCATCCA	CAGGGGAGCCATGAAGTACA
*AdipoR2*	TACCAAGGAGATTTGGAGCCC	GCCCATAAACCCTTCATCTTCC
*aco*	CAGCTAAGTTGCTTGTCTTTACCTC	CACCAAAATACAGGAATACCATAGC
*ppar*-α	GTACGGTGTGTATGAAGCCATCTT	GCCGTACGCGATCAGCAT
*fasn*	TGATGTGGAACACAGCAAGG	GGCTGTGGTGACTCTTAGTGATAA
*srebp*-*1a*	ACTTTTCCTTAACGTGGGCCT	CATCTCGGCCAGTGTCTGTT

## Supplementary Materials

The following are available online at https://www.mdpi.com/2072-6643/11/5/1133/s1, Table S1: Formula and caloric information for D12450J (Low-fat diet), Table S2: Formula and caloric information for D12451J (High-fat diet), Figure S1: Visceral fat of mice at the end of the experiment, Figure S2: Oil red staining of hepatic tissues, Figure S3: Scores for thymus and spleen indices, Figure S4: Hematoxylin and Eosin staining of colonic tissues, Figure S5: Lipid content in mice sera, Figure S6: Gut microbiota differences in mice, Figure S7: The abundances of selected bacteria after treatment.
