# Supplementary material for: A Pilot Study on Anti-Obesity Mechanisms of Kappaphycus Alvarezii: The Role of Native κ-Carrageenan and the Leftover Sans-Carrageenan Fraction
Source: Nutrients. 2019 May 21;11(5):1133. doi: 10.3390/nu11051133 (PMC6566674; doi:10.3390/nu11051133)
Supplement: Supplementary file 1 [file nutrients-11-01133-s001.pdf]

Table S1. Formula and caloric information for D12450J (Low-fat diet).

Class description	Ingredient	Grams
**Protein**	Casein, Lactic, 30 Mesh	200.00 g
**Protein**	Cystine, L	3.00 g
**Carbohydrate**	Starch, Corn	506.20 g
**Carbohydrate**	Lodex 10	125.00 g
**Carbohydrate**	Sucrose, Fine Granulated	72.80 g
**Fiber**	Cellulose	50.00 g
**Fat**	Soybean Oil	25.00 g
**Fat**	Lard	20.00 g
**Mineral**	S10026B	50.00 g
**Vitamin**	Choline Bitartrate	2.00 g
**Vitamin**	V10001C	1.00 g
	Total:	1055.00 g

**Caloric Information (Physiological Fuel Values)**	
Protein:	**20**% kcal
Fat:	**10**% kcal
Carbohydrate:	**70**% kcal
Energy Density:	**3.82** kcal/g

Table S2. Formula and caloric information for D12451J (High-fat diet).

Class description	Ingredient	Grams
**Protein**	Casein, Lactic, 30 Mesh	200.00 g
**Protein**	Cystine, L	3.00 g
**Carbohydrate**	Sucrose, Fine Granulated	176.80 g
**Carbohydrate**	Lodex 10	100.00 g
**Carbohydrate**	Starch, Corn	72.80 g
**Fiber**	Cellulose	50.00 g
**Fat**	Lard	177.50 g
**Fat**	Soybean Oil	25.00 g
**Mineral**	S10026B	50.00 g
**Vitamin**	Choline Bitartrate	2.00 g
**Vitamin**	V10001C	1.00 g
	Total:	858.10 g

**Caloric Information (Physiological Fuel Values)**	
Protein:	**20**% kcal
Fat:	**45**% kcal
Carbohydrate:	**35**% kcal
Energy Density:	**4.7** kcal/g

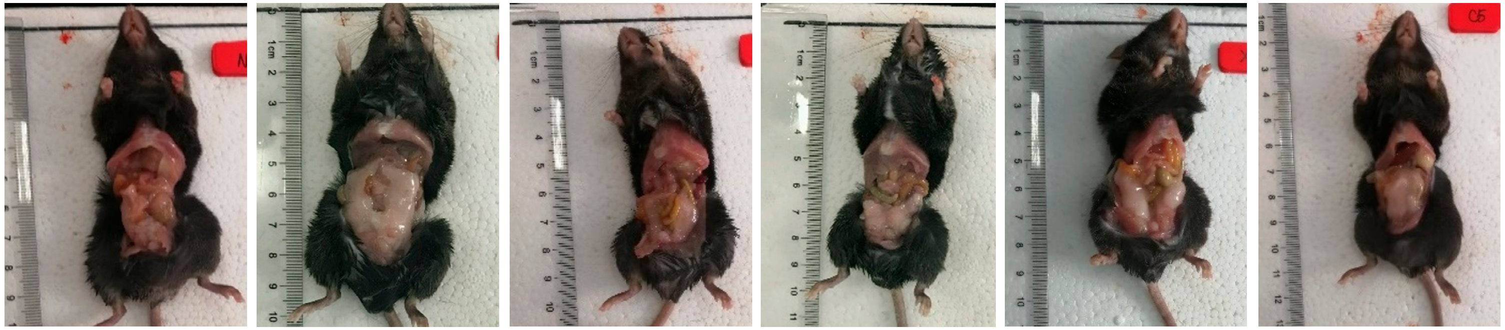

(A) (B) (C) (D) (E) (F)

Figure S1. Visceral fat of mice at the end of the experiment: (A) Normal, (B) Model, (C) Orlistat, (D) Whole Kappaphycus, (E) *Sans*-carrageenan fraction and (F) Carrageenan.

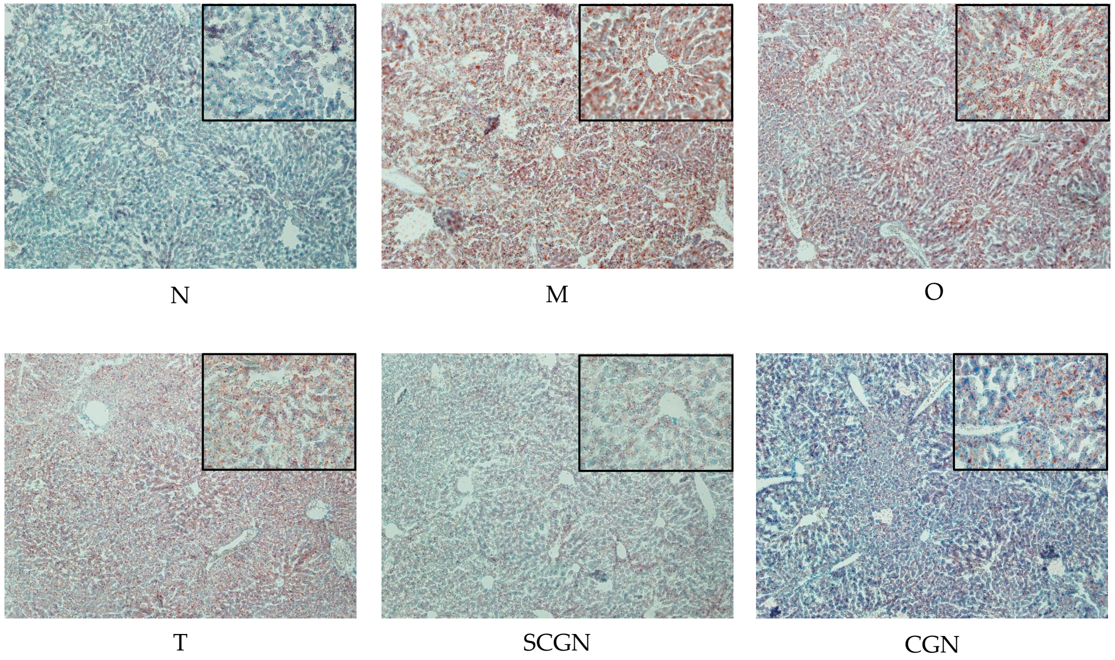

Figure S2. Oil red staining of hepatic tissues (100X magnification). Image on top right corner is the image under 400X magnification. N= Normal, M= Model, O= Orlistat, T= Whole *Kappaphycus*, SCGN= *Sans*-carrageenan fraction, CGN= Carrageenan.

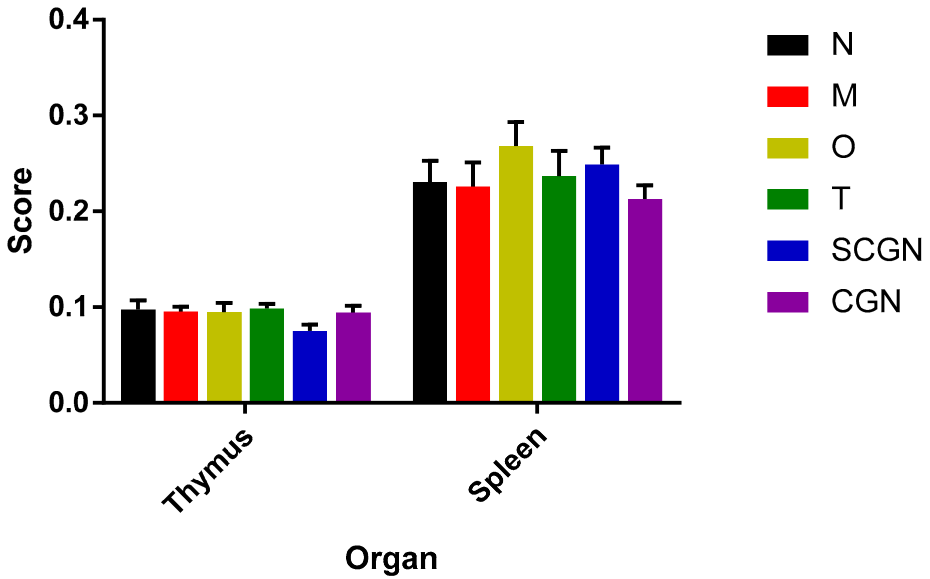

Figure S3. Scores for thymus and spleen indices. N= Normal, M= Model, O= Orlistat, T= Whole *Kappaphycus*, SCGN= *Sans*-carrageenan fraction, CGN= Carrageenan. Values are given as mean ± SEM; n= 8.

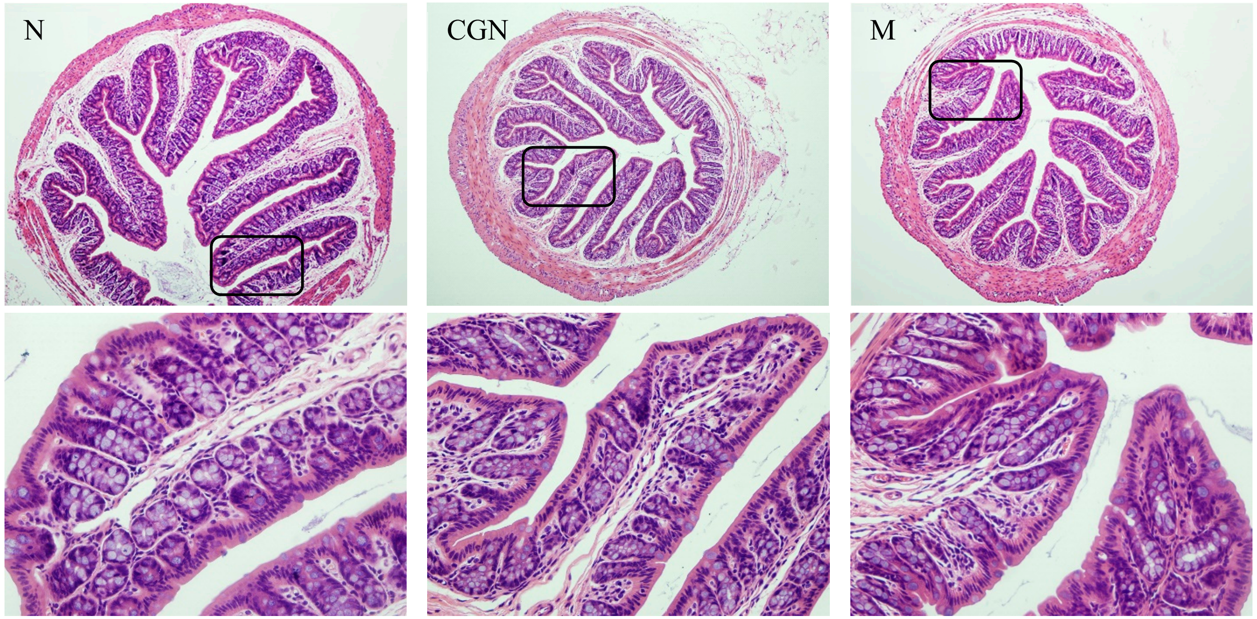

Figure S4. Hematoxylin & Eosin staining of colonic tissues of group N, CGN and M at magnification power of 40x (above) and 400x (below). N= Normal, M= Model, CGN= Carrageenan.

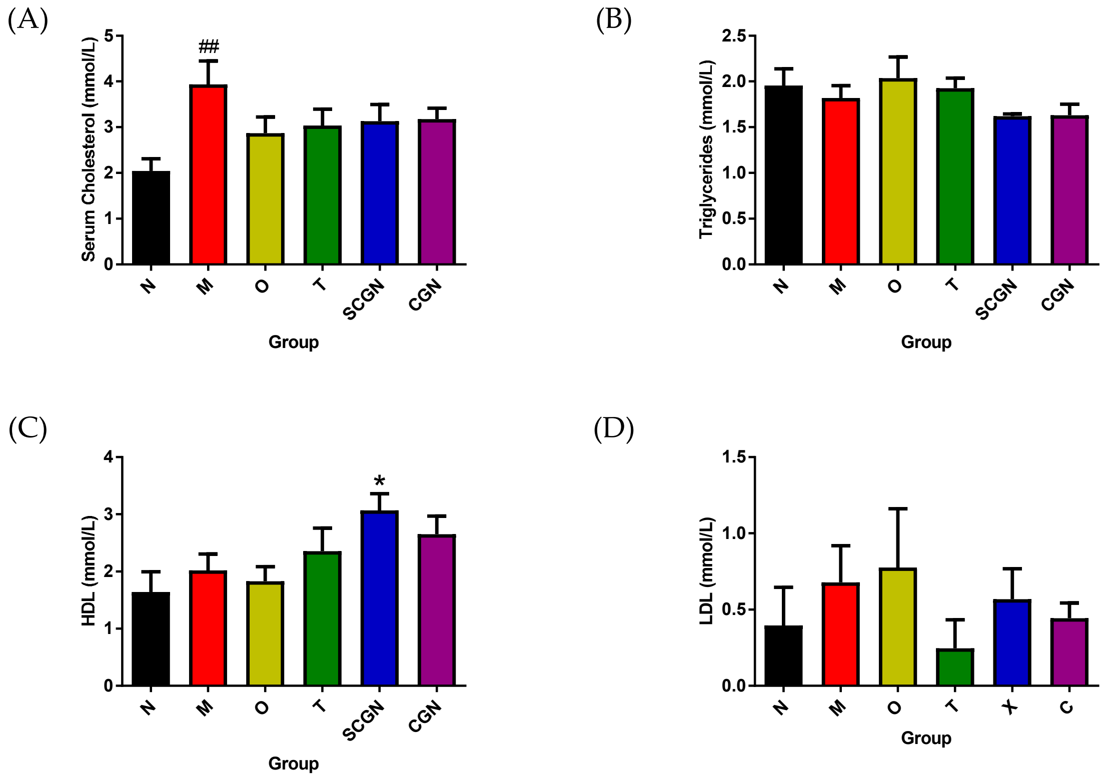

Figure S5. Lipid content in mice sera: (A) Total serum cholesterol, (B) Total serum triglycerides, (C) High-density-level (HDL) cholesterol content, (D) Low-density-level (LDL) cholesterol content. N= Normal, M= Model, O= Orlistat, T= Whole *Kappaphycus*, SCGN= *Sans*-carrageenan fraction, CGN= Carrageenan. Values are given as mean ± SEM, n= 8. # denotes significance compared to N, * denotes significance compared to M; *$p < 0.05$.

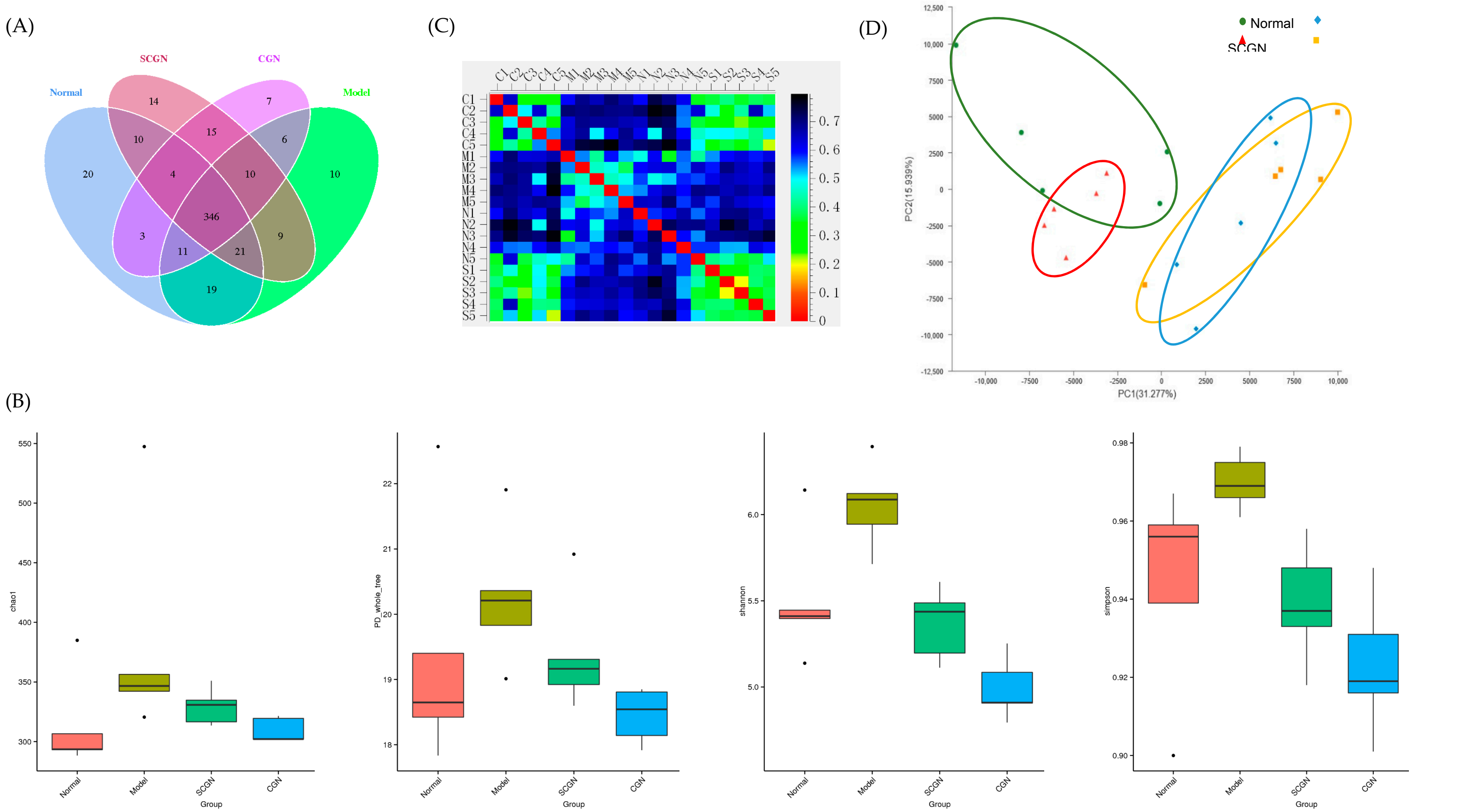

Figure S6. Gut microbiota differences in mice: (A) Venn diagram of microbiota differences, (B) Alpha diversity indices (from left: Chao1, PD whole tree, Shannon, and Simpson), (C) Bray-Curtis dissimilarity index between N, M, SCGN and CGN, (D) PCA plot of N, M, SCGN and CGN. N= Normal, M= Model, SCGN= Sans-carrageenan fraction, CGN= Carrageenan; n= 5.

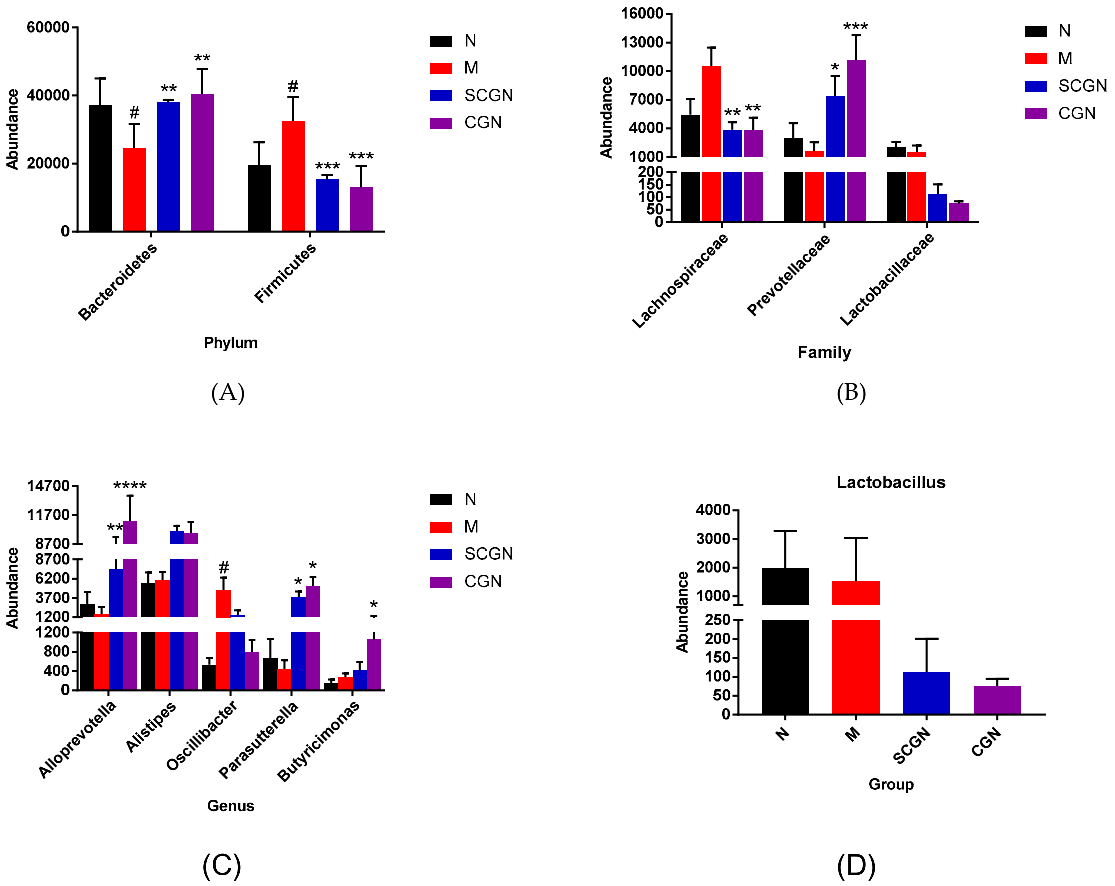

Figure S7. The abundances of selected bacteria after treatment: (A) at phylum level; (B) at Family level; (C) At genus level; (D) genus *Lactobacillus*. N= Normal, M= Model, SCGN= *Sans*-carrageenan fraction, CGN= Carrageenan. Values are given as mean ± SEM, n= 5. Statistical significances were calculated using one-way ANOVA with Tukey post-test (*p < 0.05, **p < 0.01, ***p < 0.0005, ****p < 0.0001); # denotes versus N, * denotes versus M.
